# Relationships of internet gaming engagement, history, and maladaptive cognitions and adolescent internet gaming disorder: A cross-sectional study

**DOI:** 10.1371/journal.pone.0290955

**Published:** 2023-09-08

**Authors:** Xin Wang, Yishen Liu, Harry Kwan-ching Chu, Samuel Yeung-shan Wong, Xue Yang

**Affiliations:** 1 JC School of Public Health and Primary Care, Faculty of Medicine, The Chinese University of Hong Kong, Hong Kong, China; 2 The Chinese University of Hong Kong Shenzhen Research Institute, Shenzhen, China; Yale University, UNITED STATES

## Abstract

**Objective:**

This study tested the mediation effect of maladaptive cognition of internet gaming and moderation effect of internet gaming history in the relationship between internet gaming engagement and internet gaming disorder in adolescents.

**Method:**

A total of 2,902 secondary school students were surveyed in Hong Kong from February 2021 to December 2021. The proposed moderated mediation model was tested by PROCESS.

**Results:**

Internet gaming engagement, internet gaming history and maladaptive cognition were positively associated with internet gaming disorder symptoms. Maladaptive cognition significantly mediated the association between internet gaming engagement and internet gaming disorder symptoms in both males and females. In addition, a significant interaction between internet gaming engagement and internet gaming history was detected among females but not for males, namely, the positive relationships of internet gaming engagement with maladaptive cognition and internet gaming disorder symptoms were weaker with the increased years of internet gaming.

**Conclusions:**

Our study provides a better understanding of the underlying mechanism and boundary condition in the association between internet gaming engagement and internet gaming disorder among adolescents. Preventing interventions should aim to reduce maladaptive cognition and internet gaming engagement. Interventions targeting internet gaming engagement maybe more effective among female gamers who are beginners and all male gamers.

## Introduction

### Internet gaming disorder among adolescents

With the penetration of the internet in everyday routine, internet gaming is one of the most popular internet activities among children and adolescents. Although internet gaming as a handy form of entertainment may lead to flow experience and positive affect, some adolescents engage in extensive internet gaming with associated difficulties in everyday functioning. Adolescent internet gamers may encounter gaming-associated detrimental outcomes, such as poor school performance, interpersonal relationship difficulties and health problems (e.g. problems with eyesight or hearing, sleep insufficiency, aggressive behavior and depression) [1]. Due to the significant negative consequences of excessive internet gaming, American Psychiatric Association (APA) included Internet Gaming Disorder (IGD) in the fifth edition of the Diagnostic and Statistical Manual of Mental Disorder section III (DSM-5) as a condition warranting further research in 2013. It defines IGD as “persistent and recurrent use of the Internet to engage in games, leading to clinically significant impairment or distress in 12 months [2]. In 2022, World Health Organization (WHO) officially included “Gaming disorder, predominantly online” as a subtype of gaming disorder in the International Classification of Diseases, 11th Revision (ICD-11).

A meta-analysis showed that the prevalence of adolescent IGD in Asia was 9.9%, significantly higher than that in Europe (3.9%) and Australia (4.4%) [3]. In mainland China, two studies conducted among secondary school students reported prevalence of 12.4% (N = 1200) and 13.0 (N = 2666) [4, 5]. In Hong Kong, the prevalence of IGD ranged from 6.0% (N = 1,099) to 13.0% (N = 920) [6, 7]. High prevalence of adolescent IGD (15.0%, N = 3,136) was also reported during the pandemic [8].

#### Association between internet gaming engagement and IGD

Internet gaming engagement was operationalized as time spent on internet games [9]. Since the global outbreak of the coronavirus disease 2019 (COVID-19), social distancing and self-isolation have intensified the prevalence of behavioral addictive problems in both adults and adolescents [10, 11]. Research has suggested that COVID-19 and behavioral addictions (including IGD) might constitute two pandemics that may intersect and represent a significant public health threat [12]. In Hong Kong, the hours spent on internet leisure activities increased from 0.87 h/d before the COVID-19 outbreak to 2.28 h/d averagely after the outbreak among adolescents [13]. Higher levels of engagement in internet games may lead to a higher risk of IGD. A recent longitudinal study in Chinese adolescents found that respondents who spent more than six hours on internet gaming per week had significantly higher odds (2.46 times for 6–10 h and 3.44 for > 10 h) of becoming new cases of IGD [14]. There are also researchers arguing that internet gaming engagement does not necessarily lead to IGD, as IGD needs to involve negative outcomes and deficient impulse control rather than solely excessive use [15]. People may develop profound passion and engagement with internet games, without experiencing the negative consequences [16]. Therefore, more studies are warranted to better understand the relationships between internet gaming engagement and IGD and their underlying mechanisms.

### Mediation role of maladaptive cognitions related internet gaming

Maladaptive cognitions are strong determinants in development and maintenance of addictive behaviors (e.g., gambling disorder) and psychopathology (e.g., depression) [17, 18]. Maladaptive cognitions related to internet gaming (MCIG) refer to distorted thoughts and thought processes that individuals form toward themselves and the world in the context of playing internet games [19]. King and Delfabbro [20] identified four categories of MCIG based on a systematic review: 1) beliefs about internet game reward value and tangibility (overvaluing), 2) maladaptive and inflexible rules about internet gaming behavior (maladaptive rules), 3) over-reliance on internet gaming to meet self-esteem needs (gaming self-esteem), and 4) internet gaming as a method of gaining social acceptance (gaming acceptance). Internet gamers with such maladaptive cognitions tend to use internet gaming as a coping mechanism to escape/avoid real-life issues and to gain a sense of control in the virtual world, which may increase one’s dependence to internet gaming [21, 22]. Indeed, previous studies reported that the four cognitions were significantly associated with IGD, after controlling for internet gaming time and psychological distress [23]. Kakul, and Javed also found that the correlation between overvaluation, maladaptive rules, gaming acceptance and IGD symptoms were stronger when compared with gaming self-esteem [24]. Furthermore, a study in French-speaking adults found that online gamers had more gaming related distorted cognitions than offline gamers, and the distorted cognitions were significant predictors of IGD [25].

Some evidence suggests that high internet gaming engagement may lead to MCIG. For instance, a cross-sectional study in Chinese massive multiplayer online game (MMOGs) users showed that repeated exposure to the MMOGs influenced gamers’ cognitions and feelings toward the games [26]. An experimental study found that the reduction of hours of internet gaming successfully reduced maladaptive gaming cognitions among participants with IGD [27]). The cognitive-behavioral model of pathological internet use (PIU) has been applied to explain the development and maintenance of both generalized and specific pathological internet use [19, 23]. According to the model, prolonged internet game engagement would let people expose themselves to internet gaming stimuli (e.g., sounds, images, goals) continuously. Such stimuli can serve as a positive conditioned response, which would reinforce individuals’ neuronal circuits associated with motivation and reward processing (i.e., developing MCIG) and result in continuing internet gaming. Thus, MCIG can be enhanced by persistent exposure and engagement in internet games, which in turn, contribute to the development of IGD [19]. However, no study has tested the mediating role of MCIG in the association between internet gaming engagement and IGD.

### Moderation role of internet gaming history

Internet gaming history, operationalized as the number of years a person has played internet games [28], may moderate the proposed mediation model. A long history of internet gaming may imply that the person may start to play internet games at younger age and develop habitual gaming behaviors [29]. The Uses and Gratifications theory posits that internet gamers’ prolonged internet gaming history is largely due to the gratification experiences in internet games [30]). Habitual gaming together with the prolonged gratification experiences can stabilize the cue-reactivity and craving [31], which may reinforce the association among internet gaming engagement, internet-related cognitive biases and IGD symptoms. Longer substance/non-substance use histories (e.g., smoking and gambling) or earlier initiation in adolescence maybe associated with lasting and detrimental health outcomes, such as the development of cognitive and behavioral dependence to the substance or behavior and higher rates of relapse and unsuccessful abstinence [32–34].

We only identified one empirical study and it reported a positive association between online gaming history and internet addiction [35]. This study aimed to gain empirical evidence on its roles in developing maladaptive cognition and addiction to internet gaming. It is hypothesized that a long history of internet gaming would enhance MCIG and IGD symptoms, and intensify the associations among internet gaming engagement, MCIG, and IGD symptoms.

### The present study

**Fig 1:**
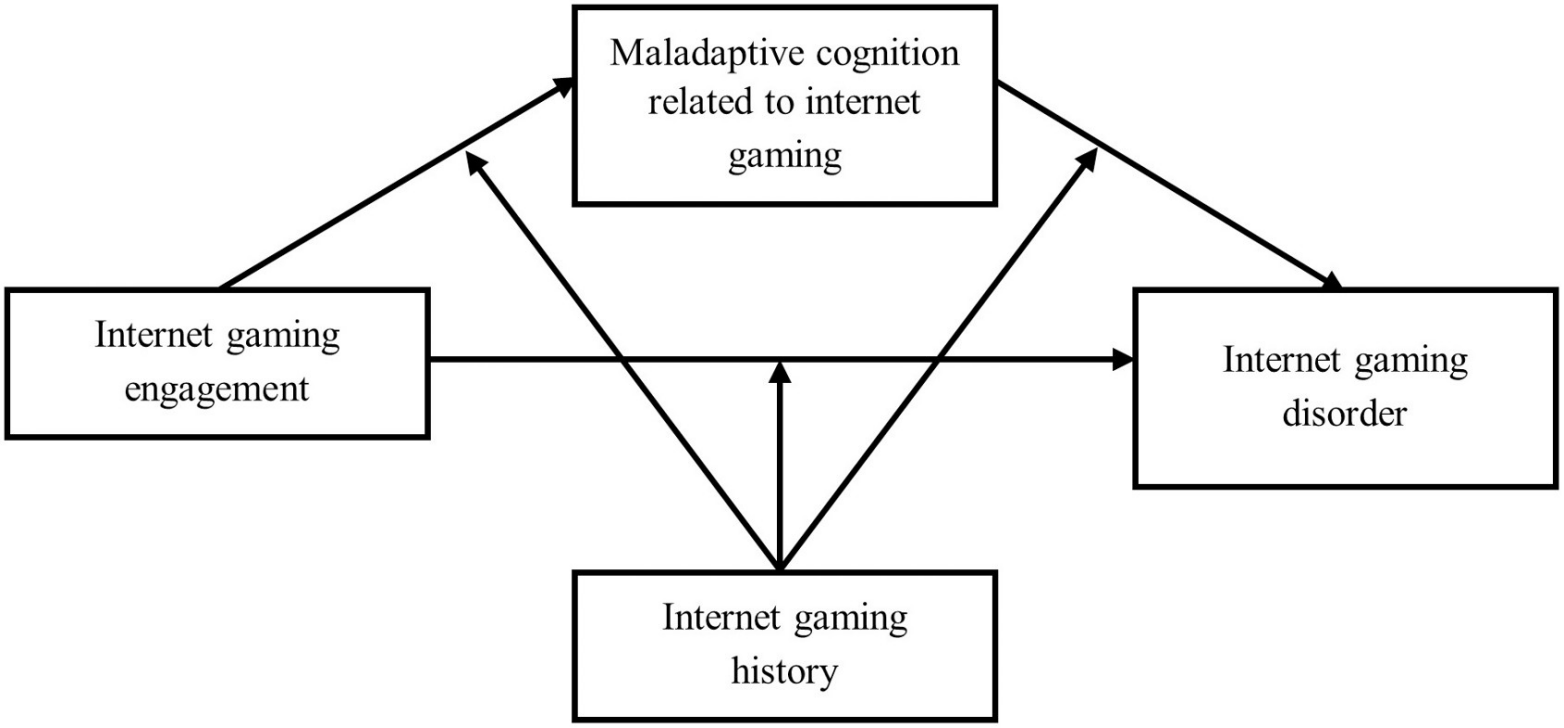
The proposed moderated mediation model.

By using a large-scale sample in Hong Kong, the current study presented the prevalence of IGD among Chines adolescents; And examined the underlying mechanism (i.e., the mediation role of MCIG and the moderation role of internet gaming history) in the association between internet gaming engagement and IGD (Fig 1). We hypothesized that internet gaming engagement would be positively associated with MCIG, which in turn would increase the risk of IGD. Also, the effects of internet gaming engagement and MCIG in the hypothesized mediation model would be strengthened among adolescents who had a long internet gaming history. Furthermore, sex differences in the associations among the variables under studied will also be explored.

## Method

### Study design and participants

A school-based survey was conducted among secondary school students in Hong Kong from February 2021 to December 2021. Twelve non-international schools were randomly selected from the Secondary School List posted by Hong Kong Education Bureau. Our experienced research staff obtained the contact information, and an invitation letter was sent to school principals to explain the significance and logistics of the study. Replacements of schools are made upon refusals. The inclusion criteria were: 1) being secondary 1–4 students; 2) providing students’ and parental consent, and 3) Chinese speaking. Secondary 5–6 students were not invited to participate in this survey because they needed to prepare for public examinations.

### Recruitment procedures

With teachers’ assistance, parents received an invitation letter and information sheet to explain the significance and logistics of the study. Parental and students’ informed consent was obtained. Participants were explained that participation was voluntary and anonymous, and rejection would not affect any right or service they would receive from the school. They were also guaranteed that only the research team could access their data. Research assistants with a training background in psychology and at least six months of interviewing experience delivered the survey in classroom settings in the absence of teachers. No incentive was given to the participants. The study procedures were carried out in accordance with the Declaration of Helsinki. Ethics approvals were obtained from the Survey and Behavioral Ethics Committee (Reference No, SBRE-18-429) and the Joint CUHK-NTEC CREC (IRB No. 2019.181) of the Chinese University of Hong Kong. All relevant data are within the manuscript and its S1 File.

### Measures

#### Internet gaming engagement

Participants were asked to report the hours spent on internet games per day in average during the past three months [9].

### Internet gaming history

Participants were asked to report the number of years they had played internet games [28, 35]. We regrouped internet gaming history into four categories: ≤ 2 years, 2–4 years, 4–6 years and >6 years [36].

#### Maladaptive cognition related to internet gaming

MCIG were measured by the 24-item Internet Gaming Cognition Scale [23]. Sample items include “I tend to feel better after playing internet games” and “When I make mistakes, lose progress, or fail in an internet game, I must reload and try again”. The items are measured using 3-point Likert scales, ranging from “*1 = do not agree” to “2 = strongly agree*”. A higher score implies a higher level of maladaptive cognition. The Chinese version has been validated in Chinese adolescents and showed satisfactory psychometric properties [37]. The scale reliability was good in the present study (Cronbach’s alpha = .91).

#### Internet gaming disorder symptoms

IGD symptoms were assessed using the DSM-5 IGD Symptoms Checklist for Adolescents (DISCA) which was developed based on the DSM-5 criteria. It consists of nine questions that assess IGD symptoms, including preoccupation, tolerance, withdrawal, unsuccessful attempts to limit internet gaming, deception or lies about internet gaming, loss of interest in other activities, use despite knowledge of harm, use for escape or relief of negative mood, and harm [38]. The response options for each item include “*yes = 1” and “no = 0*”. The overall score of the scale ranges from 0 to 9, with a higher score implying a higher level of IGD symptoms. Participants who met≥5 DSM-5 criteria were classified as having probable IGD. The Chinese version was found to have good psychometric properties among Chinese adolescents [39] (Cronbach’s alpha = .67).

#### Background factors

Background factors, including age, sex, living arrangements, family income and parental education levels, were reported by the participants.

### Statistical analysis

Descriptive statistics, including mean, standard deviation (SD) and frequency, were presented by sex. Chi-square tests and t-tests were used to compare the levels of variables between males and females. Pearson’s ^®^/Spearman’s (ρ) correlation coefficients between the independent variables (i.e., internet gaming engagement, MCIG, internet gaming history and background variables) and IGD symptoms were conducted by sex.

The mediation role of MCIG and the moderation role of internet gaming history were analyzed by Ha’es’s PROCESS macro (Model 4 and Model 59) stratified by sex. All independent variables were standardized prior to analysis. All background factors were controlled as covariates. The size of mediation effect (the proportion of mediation [PM]) was reported. The significance of the interaction term was evaluated and tested by using the change of F-values. The bootstrapping method produced 95% bias-corrected confidence intervals of these effects from 5000 resamples of the data. Confidence intervals that do not include zero means effects that are significant. Standardized regression coefficients (β), R^2^ and ΔR^2^ were also presented. Simple slope analyses were conducted for the significant interaction effects. Series mean method was used to deal with any missing values. Statistical significance was set at the .05 level. SPSS 27.0 Statistics for Windows were used for all statistical analyses. Data is available upon request.

### Sample size justification

Our primary aim was to investigate the moderation effect of internet gaming history on the proposed mediation model. In our moderated mediation model, the R square increased by interaction terms ranging from .008 - .009 among females; the effect size f^2^ was .016 - .018. A post hoc power analysis using G-Power (version 3.1) indicated that the current sample of 986 female students would provide an 86.1% power to detect an effect size f^2^ = .016 (α = .05, F-test: multiple linear regression model).

## Results

### Descriptive analyses

In total, 3354 out of 3408 students who were invited to participate in the study completed the questionnaire (response rate: 98.4%). Among them, 2902 (86.5%) internet game players were included in the data analyses. Table 1 shows the background and psychological characteristics of the participants by sex. Most of the participants were aged 13–15 years old (77.9%), living with both parents (79.7%) and male (65.8%). Males reported more hours per day and longer years of playing internet games, higher levels of MCIG, greater IGD symptoms and higher prevalence of probable IGD than females.

**Table 1:** Descriptive statistics of all variables.

	Total, *N* = 2902	Male, *N* = 1898	Female, *N* = 986	*χ*^*2*^*/t*-test
	*n* (%)/mean (*SD*)	*n* (%)/mean (*SD*)	*n* (%)/mean (*SD*)	
**Background variables**				
Age (years)				
≤12	424 (14.6)	306 (16.1)	118 (12.0)	13.46**
13	843 (29.0)	545 (28.7)	295 (29.9)	
14	880 (30.3)	548 (28.9)	331 (33.6)	
≥15	738 (25.4)	496 (26.1)	240 (24.3)	
Missing	17 (.6)	3 (.2)	2 (.2)	
Living with both parents				
Yes	2313 (79.7)	1538 (81.0)	769 (78.0)	3.80
No	571 (19.7)	356 (18.8)	215 (21.8)	
Missing	18 (.6)	4 (.2)	2 (.2)	
Family income level				
≤10,000	124 (4.3)	77 (4.1)	47 (4.8)	7.65
10,001–30,000	515 (17.7)	336 (17.7)	179 (18.2)	
>30,000	457 (15.7)	323 (17.0)	130 (13.2)	
Don’t know/ missing	1806 (62.2)	1162 (61.2)	630 (63.9)	
Father’s educational level				
Primary school or below	156 (5.4)	99 (5.2)	56 (5.7)	7.85*
Middle school	1265 (43.6)	799 (42.1)	463 (47.0)	
College or above	613 (21.1)	411 (21.7)	201 (20.4)	
Don’t know/missing	868 (29.9)	589 (31.0)	266 (27.0)	
Mother’s educational level				
Primary school or below	192 (6.6)	121 (6.4)	71 (7.2)	16.11**
Middle school	1315 (45.3)	825 (43.5)	485 (49.2)	
College or above	553 (19.1)	361 (19.0)	191 (19.4)	
Don’t know/missing	842 (29.0)	591 (31.1)	239 (24.2)	
**Internet gaming engagement**	4.68 (2.71)	5.16 (2.72)	3.75 (2.43)	14.23***
**MCIG**	12.71 (8.70)	14.04 (8.64)	10.14 (8.25)	11.86***
**Internet gaming history**				
≤ 2 years	647 (22.3)	279 (14.7)	365 (37.0)	276.6***
2–4 years	821 (28.3)	499 (26.3)	316 (32.0)	
4–6 years	741 (25.5)	537 (28.3)	198 (20.1)	
>6 years	693 (23.9)	583 (30.7)	107 (10.9)	
**IGD symptoms**	2.37 (1.96)	2.63 (1.98)	1.87 (1.81)	10.42***
**Probable IGD**				
Yes	408 (14.1)	311 (16.4)	94 (9.5)	25.24***
No	2494 (85.9)	1587 (83.6)	892 (90.5)	

Note. *SD* = Standardized deviation; IGD = Internet gaming disorder; MCIG = Maladaptive cognitions related to internet gaming.

*p < .05.

**p < .01.

***p < .001.

### Correlation analyses

As shown in Table 2, internet gaming engagement was positively correlated with MCIG (Males: r = .44, *p* < .001; Females: r = .54, *p* < .001) and IGD symptoms (Males: r = .38, *p* < .001; Females: r = .48, *p* < .001). MCIG were positively correlated with IGD symptoms (Males: r = .58, p < .001; Females: r = .67, *p* < .001). Internet gaming history was positively correlated with internet gaming engagement (Males and females: ρ = .28, p < .001), MCIG (Males: ρ = .29, *p* < .001; Females: ρ = .22, *p* < .001) and IGD symptoms (Males: ρ = .19, *p* < .001; Females: ρ = .16, *p* < .001).

**Table 2:** Correlation coefficients of all variables (*N*_male_ = 1898, *N*_female_ = 986).

	1	2	3	4	5	6	7	8	9
1. Age	-	.05	-.02	.01	.03	.02	-.05	.08*	-.07*
2. Living with parents	.08***	-	-.01	.07*	.06	.03	.01	.04	.04
3. Family income level	-.06*	-.04	-	.20***	.21***	-.03	-.04	-.01	-.03
4. Father education level	-.10***	.04	.27***	-	.66***	.01	-.01	.01	-.02
5. Mother education level	-.09***	.01	.28***	.72***	-	-.02	-.02	-.03	-.01
6. Internet gaming engagement	.13***	.08**	.01	-.03	-.05*	-	.54*** ^a^	.28***	.48*** ^a^
7. MCIG	-.03	.10***	-.03	-.05*	-.07**	.44***^a^	-	.29***	.67*** ^a^
8. Internet gaming history	.16***	.06**	.002	-.03	-.04	.28***	.22***	-	.16***
9. IGD symptoms	-.001	.10***	-.01	-.07**	-.07**	.38*** ^a^	.58*** ^a^	.19***	-

Note. IGD = Internet gaming disorder; MCIG = Maladaptive cognitions related to internet gaming. Figures in the left diagonal represent the correlations between variables among male students; those in the right diagonal represent the correlations between variables among female students.

^a^ Pear’on’s correlation coefficien^®^(*r*); others were Spear’an’s rank correlation coefficients (ρ).

*p < .05.

**p < .01.

***p < .001.

Living arrangement (ρ = .10, *p* < .001,) and education levels of farther (ρ = -.07, *p* = .004,) and mother (ρ = -.07, *p* = .003) were significantly correlated with IGD symptoms in males. Age (ρ = -.07, *p* < .041) was significantly correlated with IGD symptoms in females.

### Mediation analysis

MCIG significantly mediated the association between internet gaming engagement and IGD symptoms in both males (β_indirect_ = 0.23, 95%CI = [0.19, 0.26], PM = 59.0%) and females (β_indirect_ = 0.31, 95%CI = [0.27, 0.36], PM = 62.0%). The direct effect of internet gaming engagement on IGD symptoms remained significant in males (β_direct_ = 0.16, 95%CI = [0.12, .020]) and females (β_direct_ = 0.19, 95%CI = [0.13, 0.25]; Table 3).

**Table 3:** Testing the mediation effect of MCIG on the association between internet gaming engagement and IGD symptoms (*N*_male_ = 1898, *N*_female_ = 986).

	IGD symptoms		MCIG		IGD symptoms	
	Male	Female	Male	Female	Male	Female
Predictors	β [95%CI]	β [95%CI]	β [95%CI]	β [95%CI]	β [95%CI]	β [95%CI]
Internet gaming engagement	0.39 [0.34, .043]	0.50 [0.45, 0.56]	0.44 [0.40, 0.48]	0.57 [0.51, 0.63]	0.16 [0.12, 0.20]	0.19 [0.13, 0.25]
MCIG	-	-	-	-	0.52 [0.47, 0.56]	0.55 [0.50, 0.61]
R^2^	.16	.25	.21	.30	.36	.48
F	25.77***	23.39***	35.85***	30.06***	71.33***	58.71***

Note. IGD = Internet gaming disorder; MCIG = Maladaptive cognitions related to internet gaming; β = Standardized linear regression coefficient; 95% CI = 95% confidence interval. Regression models were adjusted for age, living arrangement, family income and parents’ education levels.

***p < .001.

### Moderated mediation model

**Fig 2:**
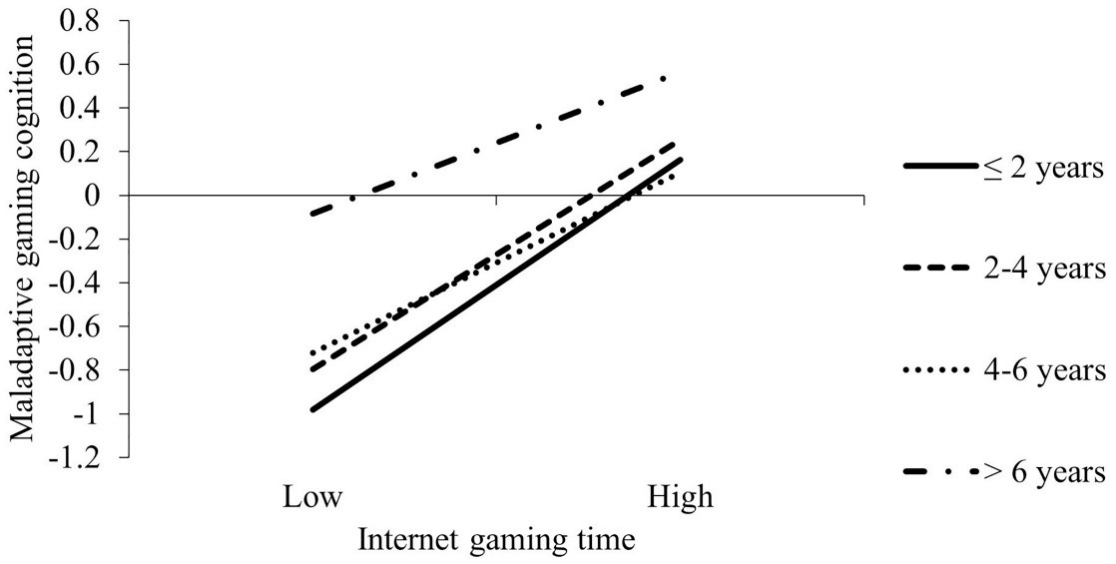
Simple slope analyses for the effect of internet game engagement on MCIG at different levels of internet gaming history among females. Note. Adjusted for age, living arrangement, family income and parents’ education levels. *** p < .001.

**Fig 3:**
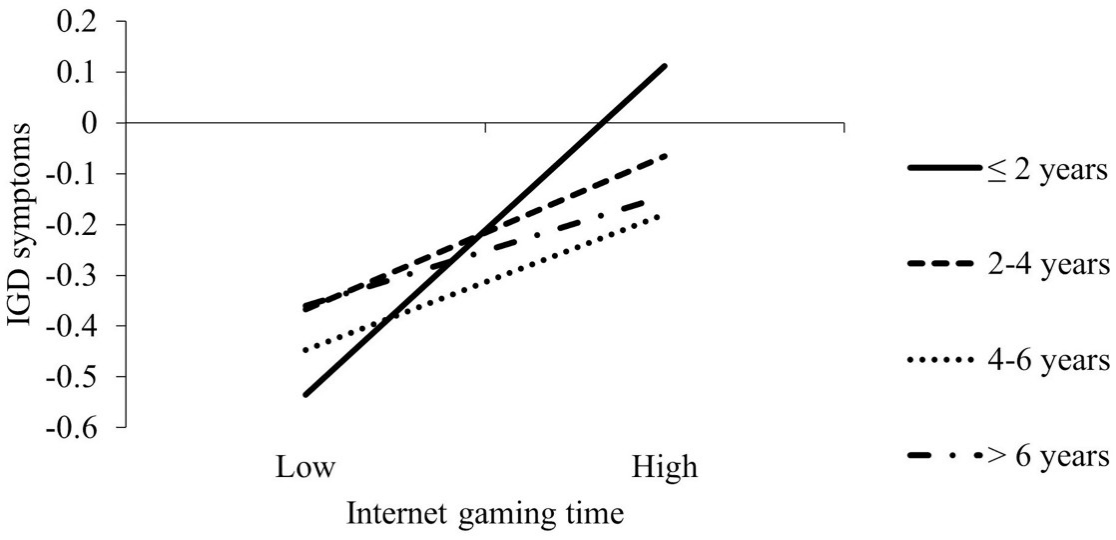
Simple slope analyses for the effect of internet game engagement on IGD symptoms at different levels of internet gaming history among females. Note. IGD = internet gaming disorder. Adjusted for age, living arrangement, family income and parents’ education levels. *** p < .001.

Table 4 presents the results of moderated mediation models by sex. In females, the association between internet gaming engagement and MCIG was negatively moderated by internet gaming history (X×W1: β = -0.05, 95%CI = [-0.21, 0.10]; X×W2: β = -0.18, 95%CI = [-0.33, -0.03]; X×W3: β = -0.28, 95%CI = [-0.45, -0.11]; *F*_change_ = 4.26, *p* = .005). Simple slope test showed that the association between internet gaming engagement and MCIG became weaker with the increased years of internet gaming (≤2 years: β = 0.64, 95%CI = [0.53, 0.74]; 3–4 years: β = 0.58, 95%CI = [0.47, 0.70]; 5–6 years: β = 0.46, 95%CI = [0.35, 0.57]; >6 years: β = 0.36, 95%CI = [0.23, 0.49]; Fig 2). The main effects of internet gaming engagement and internet gaming history on MCIG were significantly positive. MCIG was positively associated with IGD symptoms; internet gaming history did not moderate this association. The direct association between internet gaming engagement and IGD symptoms was moderated by internet gaming history (X×W1: β = -0.19, 95%CI = [-0.35, -0.03]; X×W2: β = -0.21, 95%CI = [-0.37, -0.05]; X×W3: β = -0.24, 95%CI = [-0.41, -0.07]; *F*_*c*hange_ = 3.53, p < .015). As show in Fig 3, the associations between internet gaming engagement and IGD symptoms became weaker with the increased years of internet gaming (≤2 years: β = 0.36, 95%CI = [0.25, 0.47]; 3–4 years: β = 0.17, 95%CI = [0.06, 0.28]; 5–6 years: β = 0.15, 95%CI = [0.04, 0.26]; >6 years: β = 0.12, 95%CI = [-0.002, 0.24]). The main effects of internet gaming engagement and internet gaming history on IGD symptoms were significantly positive.

**Table 4:** Testing the moderated mediation model (*N*_male_ = 1898, *N*_female_ = 986).

Predictors	MCIG		IGD symptoms	
	Male	Female	Male	Female
	β [95%CI]	β [95%CI]	β [95%CI]	β [95%CI]
Internet gaming engagement [X]	0.54 [0.40, 0.67]	0.64 [0.53, 0.75]	0.24 [0.10, 0.38]	0.36 [0.25, 0.47]
MCIG [M]	-	-	0.45 [0.33, 0.56]	0.49 [0.38, 0.59]
Internet gaming history [W]				
≤ 2 years	Ref.	Ref.	Ref.	Ref.
2–4 years [W1]	0.17 [0.04, 0.31]	0.12 [-0.02, 0.26]	0.05 [-0.08, 0.17]	-0.02 [-0.15, 0.10]
4–6 years [W2]	0.26 [0.12, 0.39]	0.04 [-0.11, 0.19]	0.12 [0.001, 0.25]	-0.17 [-0.30, -0.03]
>6 years [W3]	0.37 [0.23, 0.50]	0.55 [0.37, 0.74]	0.03 [-0.09, 0.16]	-0.12 [-0.29, 0.05]
X × W1	-0.15 [-0.31, 0.02]	-0.05 [-0.21, 0.10]	-0.004 [-0.17, 0.16]	-0.19 [-0.35, -0.03]
X × W2	-0.13 [-0.29, 0.02]	-0.18 [-0.33, -0.03]	-0.14 [-0.30, 0.02]	-0.21 [-0.37, -0.05]
X × W3	-0.15 [-0.30, 0.001]	-0.28 [-0.45, -0.11]	-0.10 [-0.25, 0.05]	-0.24 [-0.41, -0.07]
M × W1	-	-	0.09 [-0.05, 0.24]	0.15 [0.02, 0.29]
M × W2	-	-	0.09 [-0.06, 0.23]	0.03 [-0.13, 0.20]
M × W3	-	-	0.06 [-0.07, 0.20]	0.01 [-0.16, 0.17]
R^2^	.23	.34	.37	.49
F	27.56***	25.14***	45.26***	38.15***
ΔR^2^ [X × W]	.002	.008	.002	.006
F [X × W]	1.32	4.26**	2.03	3.53*
ΔR^2^ [M × W]	-	-	.001	.003
F [M × W]	-	-	0.62	2.04

Note. IGD = Internet gaming disorder; MCIG = Maladaptive cognitions related to internet gaming; β = Standardized linear regression coefficient; 95% CI = 95% confidence interval; Ref. = Reference group. Regression models were adjusted for age, living arrangement, family income and parents’ education levels.

*p < .05.

**p < .01.

***p < .001.

Among males, the main effects of internet gaming engagement and internet gaming history on both MCIG and IGD symptoms were significantly positive. However, the mediation model was not moderated by internet gaming history.

## Discussion

Using a large-scale sample of Chines adolescents in Hong Kong, the present study reported the prevalence of adolescent in Hong Kong and tested a moderated mediation model to understand the association among internet gaming engagement, history, MCIG and IGD symptoms. Results found that MCIG mediated the positive association between internet gaming engagement and IGD symptoms in both male and female adolescents. Additionally, the mediation model was moderated by internet gaming history in females not in males.

Our prevalence of IGD among internet gamers (14.1%) was higher than that reported in the Netherlands (8.5%) [40], Australia (8.1%) [41] and Spain (11.4%) [42], while it was comparable with that reported in mainland China (13.6%) [43].

Such a trend is consistent with a previous meta-analysis showing that higher prevalence estimates of IGD were found in Asia compared to other regions [3]. Measures, study designs, and cultural characteristics (e.g., social marketing, cultural values, academic stress and coping resources) may contribute to the difference in the prevalence of IGD [44, 45]. Cross-cultural research is needed to compare the prevalence and explore the causes.

MCIG was significantly positively associated with IGD symptoms in the present study, consistent with previous studies [23, 25]. It confirms the postulation of the cognitive-behavioral model of pathological Internet use (PIU) that IGD is enhanced by overvaluation of internet game rewards and over-reliance on gaming for psychosocial needs [20]. Cognitive-behavioral therapy (CBT) may be particularly useful to modify MCIG. The cognitive restructuring technique of CBT helps people to identify maladaptive internet-related beliefs and correct them with alternative beliefs [46]. Mindfulness-based intervention that enhances non-judgement may be also effective in decreasing MCIG. An intervention demonstrated that reduction in MCIG was a key therapeutic mechanism in the effects of mindfulness on IGD symptoms [47]. Mindful reappraisal practice might facilitate internet gamers to reappraise their internet gaming behaviors and reduce their automatic gaming behaviors for escapism and mood modification [47].

Furthermore, our findings support the proposed mediation model that internet gaming engagement was directly and indirectly associated with IGD symptoms through MCIG. Prolonged daily exposure to online gaming stimuli, such as gaming-related words, sounds and intricate goals with high-level rewards, alters neuronal circuits associated with motivation and reward processing, which would lead to the cognitive processing bias and the development of IGD [48]. The findings may imply that regulating internet gaming time is an effective strategy to reduce cognitive bias and prevent IGD. A recent intervention reported that the techniques monitoring and feedback for internet gaming behaviors were effective in reducing internet gaming time [49]. Also, abstinence from internet games was suggested to decrease internet gaming engagement [27]. Brailovskaia, Meier-Faust [50] investigated the effectiveness of a two-week online gaming abstinence intervention among adult gamers, reporting significant reductions in online gaming time and IGD symptoms. Parental and teachers’ monitoring, regulation and education about students’ internet gaming may help to prevent IGD [51].

The identified partial mediation effect of MCIG may imply that there exist other mechanisms between internet gaming engagement and IGD. For example, in addition to cognitive process, maladaptive emotion process (e.g., emotion irreplaceability, negative emotion anticipation) may also explain the association between internet gaming engagement and IGD symptoms [26, 52]. Playing internet games can increase enjoyment, concentration, and perceived control [53]. The hedonic satisfaction would enhance emotional connection and attachment toward internet gaming which are hardly replaced by other activities [52]. With the high level of hedonic satisfaction due to internet gaming, discontinuance and reduction in internet gaming would induce negative emotions (e.g., moodiness, nervousness, or anger) [52]. Such an emotional process may also explain the link from internet gaming engagement to IGD and can be tested in future work.

We found that male adolescents had longer internet gaming history than female adolescents, and internet gaming history was positively associated with IGD symptoms. These results are consistent with previous studies on online gaming history and internet addiction [35, 54]. The longer history may imply an earlier initiation of internet gaming. As evidence has shown, early initiation of substance use and non-substance use addictive behaviors would increase impulsivity, heighten response to reward characteristics, and weaken children’s decision-making ability [55], which increase the risk of addiction and risk behaviors [56].

It is intriguing that with the increased years of internet gaming, the impact of time spent on internet games per day on cognitions and IGD became weaker among females. However, the moderation effect was not significant among males. It may suggest that the exposure effect of internet gaming on females’ cognitive and behavioral dependence to internet gaming may reduce by years. It may be due to the sex difference in motives of internet gaming. Females tend to use it for emotional regulation, while males may rely on internet gaming to develop self-esteem [57, 58]. The emotional regulation style was found to have a stronger association with various addictive behaviors among females than males [59]. Thus, it is possible that, with the increase in internet gaming history, females’ maladaptive emotional responses were enhanced, and became the main cause of IGD. Qualitative interviews may help to illustrate the causes of the sex differences in the results. Multiple-wave longitudinal studies that investigate the developmental trajectories of behavioral, cognitive, and emotional statuses and their roles in IGD are also helpful. In addition, sex differences in the development of self-regulation may be another explanation. Boys have lower self-regulation for addictive behaviors compared to girls at the same age [60]. Early physical maturation and cultural expectation (e.g., encouraging “feminine” traits) may facilitate greater self-regulation in girls than boys [61]. Here, with the increase in internet gaming years, girls may be better at self-regulation than boys, which buffered the detrimental effect of internet gaming engagement on cognitive responses and IGD symptoms. The results may imply that preventive interventions involving internet gaming time management skills may be more effective among males than females, especially for those with longer history of internet gaming. Such interventions would also be more effective for females who are beginners of internet gaming.

### Strengths and limitation

The strengths of the study included: (1) the relatively large sample size; (2) Hayes’ Process Model 4 and 59 provided a good solution and revealed important and new insights into the associations among internet gaming engagement, MCIG, internet gaming history and IGD symptoms by sex among Chinese adolescents. However, the findings should be interpreted in the light of the following limitation. First, the current study only included the secondary 1 to 4 Chinese students. The generalizability of the study results in secondary 5–6 students or other ethnic groups should be validated in future research. Second, the study is a descriptive, cross-sectional study. A prospective study is warranted to evaluate the causal relationships among the variables. Third, self-reported measures were applied in this study. Social desirability bias may consequentially exist. Fourth, our measurement of IGD had a relatively low but acceptable internal consistency (alpha = 0.67). Future research should validate the results with other scales with more satisfactory psychometric properties (e.g., 9-item Internet Gaming Disorder Scale- Short Form (IGDS-SF9) [62, 63]. At last, adolescents’ internet gaming engagement was operationalized as the typical time devoted to internet games. Although time spent on internet gaming is often a key part of practitioner guidelines regarding engagement, future work should consider broader constructs of internet gaming engagement, such as taking mental immersion and euphoria into consideration [64, 65]. In addition to hours per day and years spent on internet gaming, other temporal and behavioral variables (e.g., initiation of internet gaming, binge internet gaming) can be explored in future work to better understand the development of adolescent IGD.

## Conclusions

This is the first study to investigate the mediation role of MCIG and the moderation role of internet gaming history in the relationship between internet gaming engagement and IGD symptoms. Results suggested that MCIG is a critical mechanism underlying the association between internet gaming engagement and IGD symptoms in both males and females. While having a long internet gaming history buffered the relationships of internet gaming engagement with MCIG and IGD symptoms only among female gamers. The findings enrich our understanding of the development and mechanisms of adolescent IGD and provide important information in designing effective interventions for preventing IGD among adolescents.

## Supporting information

S1 Checklist (DOCX)

S1 File timeIGD_PlosOne. (CSV)
